# Predictors of Recurrent Atrial Fibrillation Using Mode Switch Quantification

**DOI:** 10.4021/cr292w

**Published:** 2013-10-15

**Authors:** Phillip Ruisi, John N. Makaryus, John N. Catanzaro, Michael Ruisi, Anthony Cedrone, Amgad N. Makaryus, Erik Altman, Ram Jadonath, Stuart Beldner

**Affiliations:** a Rhode Island Hospital/Brown University, Providence, RI 02903, USA; b Hofstra North Shore-LIJ School of Medicine Department of Cardiology, North Shore University Hospital, Manhasset, NY 11030, USA; c Johns Hopkins University Department of Cardiology/Division of Electrophysiology, USA; d Beth Israel Medical Center, Department of Cardiology, First Avenue at 16th Street, New York, NY 10003, USA

**Keywords:** Atrial fibrillation, Mode switch, Cardioverter defibrillator, Permanent pacemaker

## Abstract

**Background:**

The efficacy of mode switching to predict atrial fibrillation has been established in the literature. There have been few studies investigating the incidence and clinical implication of mode switch episodes quantified from implantable cardioverter defibrillator and pacemaker interrogation. We sought to investigate the incidence of mode switch recurrence in patients with implantable cardioverter defibrillators and permanent pacemakers.

**Methods:**

Mode switch was defined as any occurrence documented during device interrogation after the date of implantation. Clinical predictors (age, gender, hypertension, diabetes, syncope, atrial fibrillation (AF)), and medications were analyzed to determine association with single and recurrent mode switch occurrences.

**Results:**

There were 21 patients experiencing a mode switch event, identified from a group of 54 patients (42 males; mean age 70 ± 12 years; mean follow-up 29.1 ± 22 months (3.4 - 81.4 months)). All but two patients were receiving medical therapy including beta blockers, statins, ace-inhibitors, and anti-arrhythmics. There were 21 subjects who experienced at least one mode switch during their follow-up and 33 subjects who never experienced a mode switch during their follow-up time. The median time to first mode switch from device implantation was 39.3 months. Risk factors individually associated with any mode switch episode included: diabetes (DM) (P < 0.04) and use of digitalis (P = 0.02). Subjects who had a history of DM were 5 times more likely to have at least one mode switch occurrence. There was a significantly higher rate of mode switch among patients who were diabetic than patients who were not (3.7 per follow-up month ± 5.3 vs. 0.98 per follow-up month ± 2.02; P = 0.02). There was a significantly higher rate of mode switch among patients who were on digitalis than those who were not (3.1 per follow-up month ± 4.3 vs. 0.73 per follow-up month ± 1.9; P = 0.02).

**Conclusion:**

The main factors associated with any mode switch are having a history of diabetes and digitalis use. Those patients who are diabetics and those on digitalis may warrant closer observation and management for the development of atrial fibrillation.

## Introduction

Typical modern dual chamber pacing devices usually combine leads for right atrial sensing and a lead for pacing in the right ventricle. “Mode switching” refers to the ability of a pacemaker to reprogram itself from a tracking to a non-tracking mode in response to atrial tachyarrhythmias. In addition, the device can revert to a tracking mode when the tachyarrhythmia terminates [1]. In this setup, the ventricle will be paced following every sensed atrial event, up to a pre-programmed maximum ventricular rate. If a paroxysmal atrial tachyarrhythmia occurs as in atrial fibrillation, then the ventricle will be pacing at this rapid unstable rate sensed from the right atrium. Therefore, the concept of mode switching entails the automatic reprogramming of a pacemaker to a mode that no longer tracks the intrinsic atrial rate [2]. The right ventricle will typically both sense and pace independent of the atrial tachyarrhythmia. The device is programmed to mode switch when it senses an atrial rate above its set threshold limit. Therefore, when the sensed atrial rate falls below the mode switching cutoff value the device assumes that the atrial tachyarrhythmia has resolved and that a more physiologic stable rhythm has been restored. At this point, the pacing mode automatically reverts to the original programming mode.

The efficacy of mode switching to predict atrial fibrillation occurrence has been well established in the literature. There have been few studies, however, investigating the incidence and clinical implication of mode switch episodes quantified from implantable cardioverter defibrillators in heart failure patients. Collection of mode switching data from implantable cardioverter defibrillators may allow for the detection of an association of atrial fibrillation with certain disease states and medication regimens. Current research has demonstrated the strong association between a mode switch event and the occurrence of atrial fibrillation. Using data culled from ICD mode switches may enhance clinical decision-making regarding optimal therapeutic regimens to manage atrial tachyarrhythmias as well as whether anticoagulation is warranted [2, 3].

## Methods

Data from fifty-four patients with implantable cardioverter defibrillators followed in our electrophysiology (EP) clinic were analyzed for interrogations that revealed a mode switch between 2006 and 2009. Data was collected on the number of mode switches and the duration of the mode switch episode for each respective patient following implantation. All mode switches in this patient cohort were a result of atrial fibrillation and/or atrial flutter at an atrial rate faster than the set threshold value for the specific device. Demographic and cardiovascular risk factor data (age, gender, hypertension, diabetes, syncope, atrial fibrillation, and any other pertinent medical history) was collected, as well as the patient’s medication record in order to determine associations with single and recurrent mode switch occurrences. After collection of data from all 54 patients, statistical analysis utilizing SPSS software was performed to assess the correlation, if any, between mode switching events and clinical and demographic predictors.

## Results

Data from 21 patients that experienced at least one mode switch event out of an initial cohort of 54 patients (42 males; mean age 70 ± 12 years; mean follow-up 29.1 ± 22 months (3.4 - 81.4 months)) was collected. All but two patients were receiving medical therapy including beta-blockers, statins, ace-inhibitors, and anti-arrhythmics. There were 33 subjects who never experienced a mode switch event during their follow-up time. The median time to first mode switch from device implantation was 39.3 months (Table 1).

**Table 1:** Patient Demographics

Patient Demographics	
Gender	Male: 17/21 (80.9%)
	Female: 4/21 (19.0%)
Age	72 ± 14 years
Average Follow-up	29.1 ± 22 months
Dyslipidemia	59.0% (12/21)
Family history of CAD	36.1% (8/21)
Hypertension	78.0% (17/21)
Diabetes mellitus	14.2% (3/21)

The risk factors individually associated with any mode switch episode included: diabetes (DM) (P < 0.04) and use of digitalis (P = 0.02). Subjects who had a history of DM were 5 times more likely to have at least one mode switch occurrence. There was a significantly higher rate of mode switch among patients who were diabetic than patients who were not (3.7 ± 5.3 events vs. 0.98 ± 2.02 events per follow-up month; P = 0.02). There was a significantly higher rate of mode switch among patients who were on digitalis than those who were not (3.1 ± 4.3 events vs. 0.73 ± 1.9 events per follow-up month; P = 0.02). Interestingly, the other more classic associations with recurrent atrial fibrillation such as hypertension, pulmonary disease, valvular disease, and atrial fibrillation itself failed to yield statistical significance (Table 2).

**Table 2:** Variables Independently Associated With Mode Switch Events

Diabetic patients	3.7 mode switches per follow-up month ± 5.3	P-value = 0.02
Non-diabetic patients	0.98 mode switches per follow-up month ± 2.02	
Patients on digoxin	3.1 mode switches per follow-up month ± 4.3	P-value = 0.02
Patients not on digoxin	0.73 mode switches per follow-up month ± 1.9	

## Discussion

Atrial fibrillation is the most common cardiac arrhythmia and has important clinical implications beyond the intrinsic effects of the tachyarrhythmia itself. Affected patients are at risk for myocardial structural deterioration due to chronic tachycardia, loss of atrioventricular synchrony, tachycardia induced cardiomyopathy with progressive dysfunction of the left atrium and left ventricle, as well as the more common risks associated with atrial fibrillation such as cerebrovascular accidents due to embolic events from atrial thrombi [4, 5]. There are many causes for atrial fibrillation with the most common being hypertension worldwide. Besides the common and classic associations with atrial fibrillation, the analyses of mode switch data can allow us to possibly identify other less thought of causes for atrial fibrillation [6].

In our study, two variables were independently associated with an increased incidence of mode switch events: diabetes and concurrent digitalis therapy. The potential implications of such findings are many. Diabetes is reaching near epidemic proportions worldwide and is an acknowledged major risk factor for cardiovascular disease events. In addition, the disease has been shown to be an independent risk factor for the development of persistent atrial fibrillation [7, 8]. Even following cardioversion, the rate of arrhythmia recurrence after the procedure is markedly higher in patients with DM. Furthermore, as a result of the neuropathic changes associated with diabetes, patients with the disease have been shown to be less aware of AF symptoms which could delay discovery and management of the arrhythmia resulting in potential increased risk of adverse events such as cerebral ischemia or tachycardia-induced cardiomyopathies [8, 9]. Further analysis into the degree of glycemic control in the patients who experienced mode switch events from those who did not is warranted. Nevertheless, given the increased risk of atrial fibrillation in patients with diabetes and the lack of reliability of symptom detection in these patients, mode switch occurrences may be especially useful in guiding therapy. On the contrary, the significant P-value associated with digitalis use and AF occurrences in our data is counter-intuitive given that digitalis is commonly used for rate control in AF patients. However, it can be argued that digitalis being an antiarrythmic itself like all antiarrythmics has a pro-arrthymic potential, which would explain the increased incidence of AF episodes in our study [9, 10].

The risks associated with poorly managed atrial fibrillation are many and include stroke, unstable rapid ventricular rates, and worsening tachycardia-related cardiomyopathies, among others. Our data has shown that diabetes and digitalis therapy are associated with an increased risk of atrial fibrillation. Patients with frequent mode switch events should be further followed to determine the clinical significance of their atrial fibrillation in terms of morbidity and mortality as well as for more focused medical therapy to manage their underlying condition, particularly diabetes. Perhaps future mode switching studies will reveal additional independent risk factors for atrial fibrillation not yet discovered. More large-scale studies are required to elucidate more risk factors potentially associated with the development of atrial fibrillation [10].

## References

[1] Israel CW (2001). [Mode-switching algorithms: programming and usefulness]. Herz.

[2] Bernstein AD, Daubert JC, Fletcher RD, Hayes DL, Luderitz B, Reynolds DW, Schoenfeld MH (2002). The revised NASPE/BPEG generic code for antibradycardia, adaptive-rate, and multisite pacing. North American Society of Pacing and Electrophysiology/British Pacing and Electrophysiology Group. Pacing Clin Electrophysiol.

[3] Kamalvand K, Tan K, Kotsakis A, Bucknall C, Sulke N (1997). Is mode switching beneficial? A randomized study in patients with paroxysmal atrial tachyarrhythmias. J Am Coll Cardiol.

[4] Chugh SS, Blackshear JL, Shen WK, Hammill SC, Gersh BJ (2001). Epidemiology and natural history of atrial fibrillation: clinical implications. J Am Coll Cardiol.

[5] Heeringa J, van der Kuip DA, Hofman A, Kors JA, van Herpen G, Stricker BH, Stijnen T (2006). Prevalence, incidence and lifetime risk of atrial fibrillation: the Rotterdam study. Eur Heart J.

[6] Psaty BM, Manolio TA, Kuller LH, Kronmal RA, Cushman M, Fried LP, White R (1997). Incidence of and risk factors for atrial fibrillation in older adults. Circulation.

[7] Haissaguerre M, Jais P, Shah DC, Takahashi A, Hocini M, Quiniou G, Garrigue S (1998). Spontaneous initiation of atrial fibrillation by ectopic beats originating in the pulmonary veins. N Engl J Med.

[8] Movahed MR, Hashemzadeh M, Jamal MM (2005). Diabetes mellitus is a strong, independent risk for atrial fibrillation and flutter in addition to other cardiovascular disease. Int J Cardiol.

[9] Potpara T, Marinkovic-Eric J, Grujic M, Radojkovic-Cirovic B, Vujisic-Tesic B, Petrovic M (2002). [Effect of diabetes mellitus in recovery and maintenance of sinus rhythm in patients with persistent atrial fibrillation]. Srp Arh Celok Lek.

[10] Sugishita K, Shiono E, Sugiyama T, Ashida T (2003). Diabetes influences the cardiac symptoms related to atrial fibrillation. Circ J.

